# Lymphatic dysfunction and impaired interstitial clearance in sarcopenic obesity: a hypothesis-generating review

**DOI:** 10.3389/fendo.2026.1876993

**Published:** 2026-07-02

**Authors:** Chunli Yu, Xinyu Zou, Rongli Dai, Yan Yang

**Affiliations:** 1 Department of Endocrinology and Metabolism, The Second Affiliated Hospital of Zunyi Medical University, Zunyi, China; 2 Department of Endocrinology and Metabolism, Affiliated Hospital of Zunyi Medical University, Zunyi, China; 3 Zunyi Medical University, Zunyi, China

**Keywords:** adipose tissue, chronic inflammation, gut–muscle axis, insulin resistance, interstitial fluid, lymphatic dysfunction, sarcopenic obesity, skeletal muscle

## Abstract

Sarcopenic obesity (SO) is characterized by excess adiposity together with reduced skeletal muscle mass and impaired muscle function, and primarily affects older adults. Its pathogenesis involves chronic low-grade inflammation, insulin resistance, ectopic lipid accumulation, mitochondrial dysfunction, and disrupted skeletal muscle homeostasis. Current models mainly explain how inflammatory and metabolic signals are generated through adipose–muscle crosstalk, but less clearly address why these signals persist within the local interstitial environment. The lymphatic system maintains interstitial homeostasis by regulating tissue drainage, mediator clearance, immune trafficking, lipid transport, and microenvironmental renewal. In SO, ageing and obesity may jointly impair lymphatic function: obesity increases inflammatory, lipid, and metabolic lymphatic stress, whereas ageing lowers baseline lymphatic reserve. These abnormalities may sustain a pro-inflammatory, pro-lipotoxic, and repair-unfavorable interstitial milieu. In this review, we summarize ageing- and obesity-associated lymphatic abnormalities and propose a clearance-centered framework in which impaired lymphatic clearance amplifies local inflammation, lipid stress, insulin resistance, gut–muscle axis disturbance, and defective skeletal muscle repair in SO. Skeletal muscle deterioration may further weaken contraction-assisted lymphatic return, potentially reinforcing impaired clearance and local inflammatory–metabolic stress. This framework complements the adipose–muscle crosstalk model and highlights testable directions for future SO-specific studies.

## 1 Introduction

With accelerated population aging and the continued rise in obesity prevalence, sarcopenic obesity (SO) has emerged as an increasingly important public health concern (1–3). SO is a complex metabolic and body-composition condition characterized by excessive adiposity together with reduced skeletal muscle mass and function. Compared with obesity or sarcopenia alone, SO is associated with poorer clinical outcomes, including reduced physical performance, falls, fractures, cardiometabolic complications, and increased mortality risk (2, 3).

The pathogenesis of SO is multifactorial and involves chronic low-grade inflammation, insulin resistance, lipotoxicity, mitochondrial dysfunction, and progressive disruption of skeletal muscle homeostasis (4–6). Existing models have largely explained SO through endocrine and metabolic crosstalk between adipose tissue and skeletal muscle, emphasizing the generation of abnormal inflammatory, adipokine, myokine, lipid-derived, and metabolic signals and their downstream effects (4–6). However, these models do not fully explain why such signals persist within the local tissue environment. In this context, interstitial clearance may be important because inflammatory mediators, lipid-related signals, immune cells, and metabolic by-products are not only produced within tissues but also transported, retained, or removed through the interstitial–lymphatic system (7, 8).

The lymphatic system is central to interstitial homeostasis because it regulates tissue drainage, mediator clearance, immune-cell trafficking, lipid transport, and microenvironmental renewal (7, 8). In obesity, adipose tissue expansion, inflammatory activation, and metabolic stress can induce structural and functional lymphatic abnormalities, including impaired lymphatic drainage, increased permeability, reduced collecting-vessel pumping, and decreased local clearance efficiency (9–12). In parallel, ageing may reduce baseline lymphatic reserve and further compromise lymphatic transport, fluid homeostasis, and clearance capacity (13–15). These two processes are particularly relevant to SO, which occurs primarily in older adults with excess adiposity.

**Figure 1:**
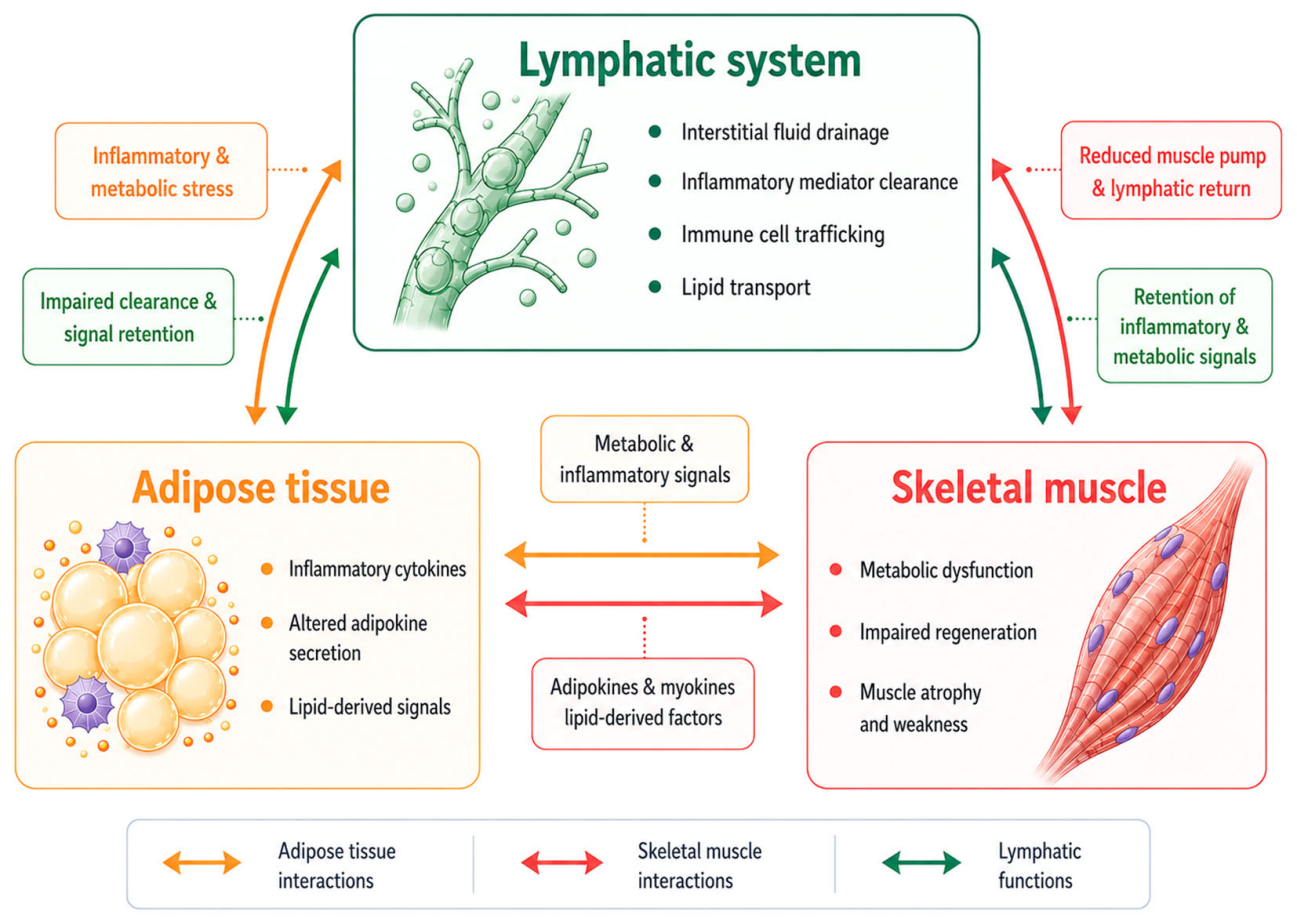
Proposed interaction network among adipose tissue, the lymphatic system, and skeletal muscle in sarcopenic obesity. This conceptual framework illustrates the bidirectional relationships among adipose tissue, the lymphatic system, and skeletal muscle. Dysfunctional adipose tissue may increase inflammatory and metabolic stress, whereas impaired lymphatic function may lead to impaired clearance and signal retention. Skeletal muscle deterioration may further reduce contraction-assisted lymphatic return, thereby reinforcing local inflammatory–metabolic imbalance. This framework complements the established adipose–muscle crosstalk model by highlighting lymphatic regulation as a potential modulator of tissue microenvironmental homeostasis.

**Figure 2:**
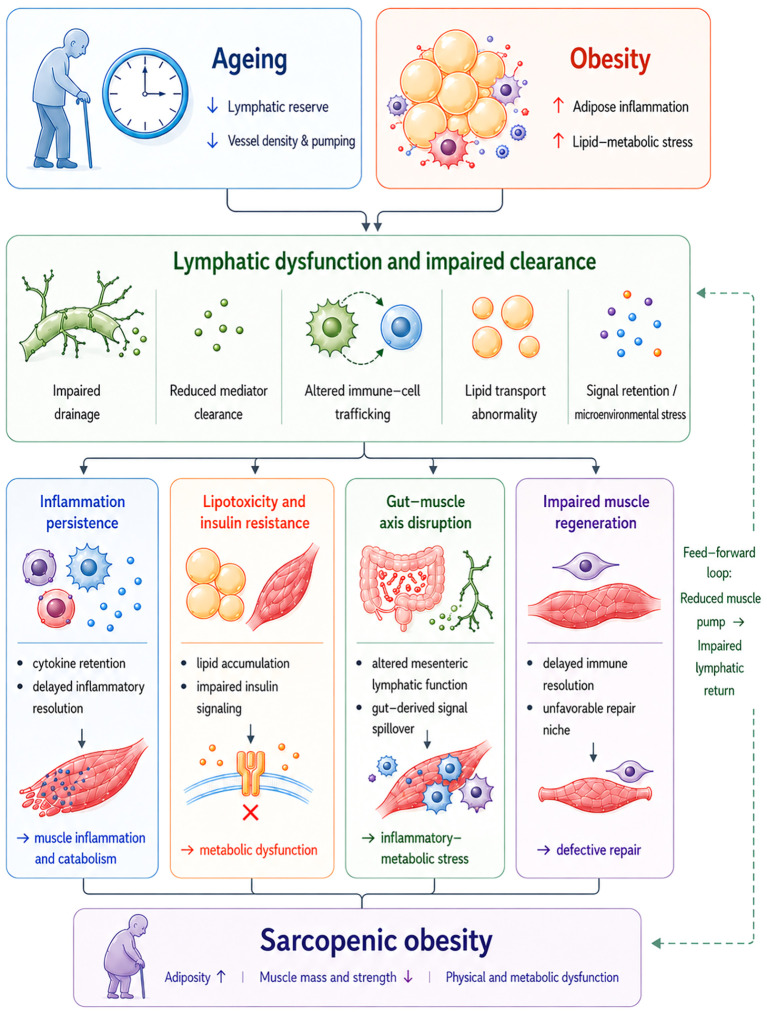
Proposed mechanisms by which ageing- and obesity-associated lymphatic dysfunction may amplify inflammatory–metabolic stress in sarcopenic obesity. Ageing may reduce lymphatic reserve, vessel density, and pumping capacity, whereas obesity may increase adipose inflammation and lipid–metabolic stress. These changes may converge on lymphatic dysfunction and impaired interstitial clearance, leading to impaired drainage, reduced mediator clearance, altered immune-cell trafficking, abnormal lipid transport, and signal retention. The resulting disrupted microenvironment may amplify SO-related pathology through inflammation persistence, lipotoxicity and insulin resistance, possible gut–muscle axis disruption, and impaired skeletal muscle repair. These pathways are proposed as hypothesis-generating mechanisms and require validation in SO-specific experimental and clinical studies. The dashed feedback loop indicates that skeletal muscle deterioration may further reduce contraction-assisted lymphatic return, thereby reinforcing lymphatic dysfunction and impaired interstitial clearance.

This review therefore focuses on ageing- and obesity-associated lymphatic abnormalities not simply as vascular or drainage defects, but as disturbances in interstitial clearance that may reshape the inflammatory–metabolic environment surrounding adipose tissue and skeletal muscle in SO. We propose that lymphatic dysfunction may represent a stress-amplifying process that prolongs local inflammatory and metabolic signal retention, thereby complementing the conventional adipose–muscle crosstalk framework. Importantly, this framework should not be interpreted as proposing lymphatic dysfunction as an independent initiating cause of muscle loss. Rather, impaired clearance may interact with adipose tissue dysfunction, skeletal muscle deterioration, and reduced contraction-assisted lymphatic return in a bidirectional feed-forward loop. **Figure 1** illustrates the proposed adipose tissue–lymphatic system–skeletal muscle interaction network, and **Figure 2** summarizes the downstream mechanisms by which impaired lymphatic clearance may amplify inflammatory–metabolic stress and muscle dysfunction.

## 2 Sarcopenic obesity: a state of chronic inflammatory–metabolic imbalance

SO is a heterogeneous body-composition disorder in which excess adipose tissue coexists with reduced skeletal muscle mass and impaired muscle function (5, 16, 17). Its diagnosis should not rely on body mass index alone, but should consider adiposity, muscle mass, and muscle function together, consistent with the European Society for Clinical Nutrition and Metabolism (ESPEN) and European Association for the Study of Obesity (EASO) consensus, as well as the European Working Group on Sarcopenia in Older People 2 (EWGSOP2) emphasis on muscle strength (16, 17).

In SO, adipose tissue, the lymphatic system, and skeletal muscle may be functionally interconnected within the local inflammatory–metabolic microenvironment. Dysfunctional adipose tissue can increase inflammatory cytokines, adipokine imbalance, lipid-derived signals, and metabolic stress, thereby increasing the burden on local lymphatic vessels (4, 6, 9–12, 18, 19). Impaired lymphatic clearance may then allow inflammatory and lipid-related signals to persist and affect both adipose tissue and skeletal muscle (7, 8, 20, 21). Conversely, skeletal muscle contraction can support lymphatic return through a muscle-pump effect, whereas loss of muscle mass and reduced contractile activity may further weaken lymphatic return and contribute to local signal retention (22, 23).

Mechanistically, SO should not be viewed merely as a static phenotype of abnormal body composition. Rather, it reflects a dynamic inflammatory–metabolic condition in which adipose tissue dysfunction, skeletal muscle impairment, and local microenvironmental imbalance reinforce each other (4–6). Within this context, impaired interstitial clearance may provide an additional mechanism through which inflammatory and metabolic signals persist within the local tissue environment and contribute to sustained adipose–muscle dysfunction.

## 3 Ageing- and obesity-associated lymphatic remodeling: potential contributors to interstitial inflammatory–metabolic imbalance

Both obesity and ageing can impair lymphatic function, and their combined effects may jointly contribute to the inflammatory–metabolic imbalance of sarcopenic obesity. Obesity is closely associated with lymphatic dysfunction (24, 25). Adipose tissue expansion, chronic low-grade inflammation, metabolic stress, and increased lipid burden can impose sustained stress on lymphatic vessels, leading to structural remodeling, impaired barrier integrity, reduced collecting lymphatic pumping, and defective local clearance (26–28). In parallel, ageing may reduce baseline lymphatic reserve through decreased lymphatic vessel density, diminished collecting-vessel pumping, impaired macromolecular clearance, and downregulation of VEGF-C/VEGFR3 signaling (13–15). These ageing-related changes may increase vulnerability to obesity-associated lymphatic injury in older adults. Beyond peripheral adipose tissue, lymphatic abnormalities in mesenteric and visceral adipose regions may have particularly important metabolic consequences because these regions participate directly in intestinal lipid absorption, antigen delivery, and metabolic signal transport (29, 30). Therefore, ageing- and obesity-associated lymphatic alterations should be considered not only as consequences of obesity-related lymphatic overload or ageing-related reserve loss, but also as potential contributors to impaired interstitial renewal, prolonged signal retention, and inflammatory-metabolic imbalance (24, 26).

### 3.1 Mechanisms of lymphatic injury driven by adipose tissue inflammation and metabolic stress

Obesity-associated lymphatic injury reflects the combined effects of adipose tissue expansion, chronic low-grade inflammation, metabolic stress, and lipotoxicity (18, 24–28). Functionally, these changes can be organized into lymphatic endothelial injury, barrier dysfunction with increased permeability, reduced lymphatic capillary density, weakened collecting lymphatic pumping, impaired immune-cell trafficking, and defective interstitial clearance (11, 26–28, 31–34). Together, they represent a shift from efficient tissue drainage and immune transport toward impaired clearance and local inflammatory–metabolic retention.

This functional impairment is relevant to SO because reduced lymphatic drainage and defective interstitial clearance may allow inflammatory and lipid-related signals, cellular debris, and metabolic by-products to persist within adipose tissue and skeletal muscle-associated microenvironments (7, 8, 21, 35). This altered microenvironment may provide a permissive background for chronic inflammation, lipotoxic stress, and impaired metabolic signaling. However, direct evidence showing that obesity-associated lymphatic dysfunction drives skeletal muscle deterioration in SO remains limited. Therefore, this link should be interpreted as a hypothesis-generating mechanism requiring validation in SO-specific models and clinical populations.

### 3.2 Specific alterations in mesenteric and visceral adipose lymphatics

Compared with subcutaneous adipose tissue, visceral adipose tissue, particularly mesenteric adipose tissue, is more closely connected to the intestinal lymphatic system and is surrounded by an extensive lymphatic network (36). Lacteals and mesenteric lymphatic vessels participate in the absorption, drainage, and transport of dietary lipids, lipid-soluble molecules, and gut-derived antigens (29). The lymphatic system also contributes to lipid homeostasis through intestinal lipid transport and peripheral cholesterol transport (29, 37–39). These functions provide a potential link between adipose tissue, lymphatic clearance, and skeletal muscle metabolic stress in SO.

In obesity, increased gut-derived lipid burden may alter mesenteric lymph composition and expose lymphatic endothelial cells to a prolonged lipid-rich environment. Experimental studies suggest that lipid overload and lymphatic endothelial defects can disrupt lacteal or collecting-vessel integrity and are associated with visceral obesity, insulin resistance, and impaired trafficking of lipids, lipid-soluble molecules, and antigens to mesenteric lymph nodes (30, 40–42). Thus, mesenteric and visceral adipose-associated lymphatic abnormalities are not merely local drainage defects. By altering lipid transport, lymph flow, and antigen delivery, they may reshape the surrounding interstitial microenvironment and provide a potential entry point into the gut–adipose tissue–muscle axis. However, whether these abnormalities directly contribute to skeletal muscle metabolic stress in SO remains uncertain. Therefore, the available evidence should be interpreted cautiously and summarized according to evidence strength and relevance to SO in **Table 1**.

**Table 1:** Evidence strength and SO relevance of the proposed lymphatic–interstitial clearance mechanisms in sarcopenic obesity.

Proposed pathway	Main supporting evidence	Relevance to the SO framework	Evidence strength
Chronic inflammation and impaired inflammatory clearance	Obesity and inflammation-related lymphatic studies show impaired lymphatic drainage, increased permeability, reduced collecting-vessel pumping, perilymphatic inflammatory cell accumulation, and delayed inflammatory mediator clearance (8, 21, 24–28, 31–35, 43–48).	Supports the possibility that impaired lymphatic clearance may prolong inflammatory mediator retention and sustain a pro-inflammatory adipose and skeletal muscle microenvironment in SO.	Limited indirect evidence; not SO-specific.
Lipid accumulation, lipid infiltration, and insulin resistance	Lymphatic defects, lymphedema, and obesity-related lymphatic dysfunction are associated with adipose expansion, adipocyte hypertrophy, fibrosis, altered lipid handling, and insulin resistance-related metabolic abnormalities (12, 49–65).	Provides the relatively strongest indirect support for the framework, especially at the adipose tissue level. However, whether lymphatic dysfunction directly promotes skeletal muscle lipid infiltration or insulin resistance in SO remains unclear.	Moderate indirect evidence; strongest among the proposed pathways, but mainly adipose-tissue based.
Gut–lymphatic–muscle axis	Intestinal and mesenteric lymphatics regulate dietary lipid absorption, antigen delivery, immune surveillance, mesenteric adipose tissue homeostasis, microbiota-related lacteal regulation, and gut-derived inflammatory signaling, including lipid-associated LPS transport under specific conditions (29, 30, 40, 42, 66–72).	Supports a biologically plausible but unproven link between intestinal lymphatic dysfunction, mesenteric adipose inflammation, gut-derived inflammatory signaling, and skeletal muscle metabolic stress.	Weak indirect evidence; hypothesis-generating and not SO-specific.
Skeletal muscle repair microenvironment and intramuscular lymphatics	Muscle repair requires inflammation resolution, immune-cell transitions, and muscle stem cell (MuSC) activation. Lymphatics regulate tissue fluid homeostasis, inflammatory mediator clearance, metabolic waste drainage, and immune-cell trafficking. Intramuscular lymphatics remodel during unloading and injury recovery (21, 23, 73–80).	Suggests that impaired lymphatic clearance may contribute to a repair-unfavorable skeletal muscle microenvironment, but evidence is derived mainly from injury, unloading, and general lymphatic models.	Weak; hypothesis-generating and indirect for SO.
Direct SO-specific lymphatic evidence	Direct studies assessing lymphatic structure, lymphatic function, or interstitial clearance in SO patients or SO-specific animal models remain lacking.	Identifies the major knowledge gap and justifies the hypothesis-generating nature of the review.	Very limited; major evidence gap.

Evidence strength was qualitatively graded according to the directness, consistency, and disease relevance of the available evidence.

### 3.3 Ageing-related decline in lymphatic reserve and its synergy with obesity

Ageing-related lymphatic decline may provide an additional vulnerability factor in the proposed SO framework. Independent of adiposity, ageing has been associated with structural and functional deterioration of the lymphatic system, including reduced lymphatic vessel density, diminished collecting-vessel pumping, impaired lymph transport, increased permeability, defective macromolecular clearance, and altered VEGF-C/VEGFR3 signaling (13–15, 43). These changes suggest that older adults may have a lower baseline lymphatic reserve before the onset or progression of obesity-related metabolic stress. Importantly, ageing-related lymphatic dysfunction is not merely a structural abnormality. Da Mesquita and colleagues showed that impaired meningeal lymphatic drainage in aged mice had functional consequences and could be improved by VEGF-C-mediated lymphatic restoration (14). Comparable age-related deterioration has also been described in peripheral lymphatic beds, including aged skin lymphatic vessels and lymphatic collectors (13, 15, 43).

In the context of sarcopenic obesity, ageing and obesity may therefore act through partly distinct but convergent pathways. Obesity increases inflammatory, lipid, and metabolic burden on lymphatic vessels, whereas ageing reduces lymphatic reserve and repair capacity. Their combination may increase susceptibility to impaired interstitial clearance, prolonged inflammatory and metabolic signal retention, and defective microenvironmental renewal. Thus, ageing-related lymphatic decline may synergize with obesity-associated lymphatic injury and provide a biologically plausible contributor to sustained inflammatory–metabolic imbalance in older adults with excess adiposity. Given the role of mesenteric and visceral adipose lymphatics in lipid transport and antigen delivery, whether ageing further aggravates lymphatic abnormalities in these regions remains an important question for future SO-specific studies (29, 30).

## 4 Proposed mechanisms linking ageing- and obesity-associated lymphatic abnormalities to sarcopenic obesity

Ageing- and obesity-associated lymphatic abnormalities may converge on a common functional consequence: impaired interstitial drainage and clearance, together with defective microenvironmental renewal. Obesity may increase inflammatory, lipid, and metabolic lymphatic burden, whereas ageing may lower baseline lymphatic reserve. In this framework, lymphatic dysfunction may amplify pre-existing inflammation, lipotoxic injury, insulin resistance, and impaired repair by prolonging tissue exposure to retained inflammatory and metabolic stressors. Conversely, progressive loss of skeletal muscle mass and contractile activity may weaken the skeletal-muscle pump that supports lymphatic return, thereby aggravating lymph stasis and local signal retention. The proposed model should therefore be interpreted as a bidirectional feed-forward loop rather than a strictly one-way pathway.

Because direct SO-specific evidence remains limited, the following mechanisms should be interpreted according to their evidence strength rather than as equally established pathways. The lymphatic dysfunction–lipid accumulation–insulin resistance pathway has relatively stronger indirect support, mainly from obesity, adipose tissue, lymphedema, and lymphatic-defect models. The chronic inflammation pathway has limited indirect support. The gut–muscle axis and impaired skeletal muscle repair pathways remain more speculative and should be considered emerging hypotheses primarily extrapolated from intestinal lymphatic biology, microbiota studies, and muscle injury or unloading models. The relative strength and SO relevance of the available evidence are summarized in **Table 1**, and related testable predictions are presented in **Box 1**.

Box 1 **Priority testable predictions of the clearance-centered hypothesis in sarcopenic obesity**

Prediction 1. Sarcopenic obesity should be associated with measurable lymphatic abnormalities beyond obesity or ageing alone.

Patients or animal models with SO should exhibit quantifiable lymphatic deficits compared with age- and obesity-matched non-sarcopenic controls. Measures may include lymphatic vessel density, tracer drainage efficiency, lymphatic permeability, collecting-vessel pumping, macromolecular clearance, or expression of lymphatic markers such as LYVE-1, PROX1, VEGFR3, and podoplanin. This prediction is based mainly on indirect evidence from obesity- and ageing-related lymphatic studies and requires direct validation in SO.

Prediction 2. Restoring lymphatic function should attenuate local inflammatory–metabolic stress and skeletal muscle deterioration.

Interventions that improve lymphatic drainage, barrier integrity, or interstitial clearance should reduce inflammatory mediator retention, intramuscular lipid accumulation, and insulin-signaling defects, and should improve muscle outcomes, including fiber cross-sectional area, grip strength, physical performance, and atrophy-related markers such as MuRF1 and Atrogin-1. This prediction is mechanistically plausible but requires SO-specific experimental testing.

### 4.1 Impaired lymphatic clearance and persistent chronic inflammation

In obesity-associated lymphatic dysfunction, persistent inflammation may reflect not only increased production of pro-inflammatory signals, but also delayed clearance of retained mediators, cellular debris, and metabolic waste. Impaired lymphatic clearance may therefore convert transient inflammatory disturbances into sustained low-grade inflammation, thereby amplifying local microenvironmental imbalance in SO.

Chronic low-grade inflammation in adipose tissue contributes not only to obesity-related metabolic disease and distant organ complications, but also to the pathogenesis of SO (18). Interstitial fluid provides the local environment through which inflammatory mediators, metabolites, and immune cells move and interact, whereas the lymphatic system supports the removal of these components, promotes inflammation resolution, and restores tissue homeostasis (8, 35). When lymphatic drainage is impaired, inflammatory mediators, cellular debris, and metabolic by-products may persist within the interstitial space and sustain local feed-forward inflammatory loops that delay inflammation resolution and tissue repair (8, 35, 44). Ageing-related reductions in lymphatic reserve may further weaken inflammatory mediator clearance in older adults, making obesity-associated inflammatory signals more likely to persist within the local interstitial environment (13–15, 43). Experimental evidence also indicates that blockade of VEGF-C/VEGFR3 signaling can aggravate inflammation, whereas enhanced lymphangiogenesis and improved lymphatic function may attenuate inflammatory responses (8). In obesity, lymphatic dysfunction is frequently associated with perilymphatic inflammatory cell accumulation, reduced collecting lymphatic pumping, increased lymphatic leakage, and aggravated local inflammatory injury (25, 32, 34, 45). These changes suggest that ageing- and obesity-associated lymphatic impairment may sustain a pro-inflammatory adipose and skeletal muscle microenvironment by limiting the clearance of inflammatory mediators and metabolic by-products.

A persistent pro-inflammatory adipose and skeletal muscle microenvironment may further disrupt skeletal muscle metabolic homeostasis and functional maintenance. Perimuscular adipose tissue and infiltrating immune cells can continuously release pro-inflammatory mediators, including TNF-α, IL-6, and IL-1β (46, 47). These cytokines can activate MAPKs, IKK/NF-κB, and JAK/STAT3 signaling, thereby impairing IRS-1-mediated insulin signaling and upregulating E3 ubiquitin ligases such as MAFbx/atrogin-1 and MuRF1 (46–48). Sustained inflammation may also suppress Akt-mediated anabolic signaling while enhancing ubiquitin-proteasome-dependent muscle protein degradation, thereby potentially promoting skeletal muscle atrophy and functional decline (46, 47). As muscle mass and strength decline, the auxiliary role of skeletal muscle contraction in lymphatic return may also be weakened, further aggravating local lymph stasis and inflammatory signal retention (22, 23). This supports a feed-forward interpretation in which impaired lymphatic clearance may prolong inflammatory exposure of skeletal muscle, while progressive muscle deterioration may in turn reduce contraction-assisted lymphatic return. These observations do not prove that impaired lymphatic clearance directly causes muscle loss in SO, but they indicate that defective clearance and skeletal muscle decline may reinforce each other within an already established catabolic environment.

### 4.2 Impaired lymphatic clearance exacerbates abnormal fat deposition, lipid infiltration, and insulin resistance

Among the proposed mechanisms discussed in this review, the link between lymphatic dysfunction and abnormal fat accumulation is supported by relatively stronger evidence than the link between lymphatic dysfunction and SO-specific muscle loss. This distinction is important because lymphatic defects and lymphedema have repeatedly been associated with local adipose expansion, adipocyte hypertrophy, and lipid metabolic dysregulation, suggesting that lymphatic abnormalities may actively disrupt adipose tissue homeostasis rather than merely accompany obesity. In older adults, reduced lymphatic reserve may limit the ability to compensate for obesity-related lipid and inflammatory overload, thereby increasing the likelihood that impaired lymphatic clearance contributes to sustained lipid and inflammatory exposure in adipose tissue and the skeletal muscle microenvironment (13–15, 43).

Genetic studies have shown that Chy and Prox1^+^/^−^ mice develop adipose tissue expansion and metabolic abnormalities because of lymphatic developmental or functional defects (49, 50). Clinically, advanced lymphedema is also frequently accompanied by chronic adipose deposition and adipocyte hypertrophy (51). Lymph-related culture conditions can enhance adipogenic activity in adipose-derived stem cells and upregulate adipogenic genes, including PPARγ, C/EBPα, FABP4, and LPL (52, 53). These studies provide a relatively direct adipose-tissue basis for considering lymphatic dysfunction in obesity-related fat accumulation. Under obese conditions, further lymphatic injury can aggravate subcutaneous fat deposition, inflammatory cell infiltration, and fibrosis (54). Impaired lymphatic clearance may therefore expose the microenvironment surrounding skeletal muscle to sustained lipid and inflammatory stress, potentially facilitating lipid infiltration, lipotoxicity, and insulin resistance.

Abnormal lipid deposition in skeletal muscle represents an important link between impaired local lipid handling and muscle metabolic injury. Increased intramyocellular lipid content is associated with reduced insulin sensitivity, impaired muscle fiber contractile function, and decreased strength (55, 56), while increased intermuscular adipose tissue is closely related to insulin resistance, muscle dysfunction, and mobility limitation (57, 58). Persistent lipid exposure can induce the accumulation of lipotoxic intermediates, such as diacylglycerol and ceramide (59), which further aggravate skeletal muscle insulin resistance by inhibiting IRS-1/PI3K/Akt signaling and reducing GLUT4 translocation and glucose uptake (60–63). Reduced Akt activity can also suppress mTOR-mediated protein synthesis and activate FoxO-related catabolic signaling, thereby potentially promoting muscle protein degradation and skeletal muscle atrophy (64, 65). Although the adipose-tissue link between lymphatic dysfunction and abnormal fat accumulation is supported by relatively direct evidence, whether this relationship extends to skeletal muscle lipid infiltration and insulin resistance in SO remains an important question for future studies.

### 4.3 Emerging hypotheses and future directions: gut–muscle axis and skeletal muscle repair

Compared with lipid accumulation and chronic inflammation, these two pathways remain more speculative and should be considered emerging hypotheses. The gut–lymphatic–muscle axis is biologically plausible because intestinal lymphatics connect dietary lipid absorption, antigen delivery, immune surveillance, mesenteric adipose tissue homeostasis, and systemic metabolic regulation (29, 30). In obesity, lacteal and mesenteric lymphatic abnormalities may alter gut-derived lipid transport, increase lymphatic leakage or reflux, promote mesenteric adipose inflammation, and contribute to systemic inflammatory–metabolic stress (30, 40, 42). These changes may be relevant to SO because mesenteric adipose inflammation, altered lipid handling, and systemic metabolic stress can potentially influence skeletal muscle insulin sensitivity, lipid exposure, and inflammatory tone.

Gut microbiota may also influence lacteal integrity and lipid absorptive function through VEGF-C-related regulation (66–68). Thus, dysbiosis-related disruption of intestinal lymphatic homeostasis could provide a possible link between gut-derived metabolic signals and the inflammatory–metabolic environment relevant to skeletal muscle. With respect to gut-derived lipopolysaccharide (LPS), available evidence indicates that LPS can associate with chylomicrons and enter mesenteric lymph under lipid-rich conditions, whereas portal venous transport appears to account for the majority of absorbed LPS (69, 70). LPS-related signaling may affect skeletal muscle inflammation, insulin signaling, and catabolic responses (71, 72), but the specific contribution of intestinal lymphatic transport to systemic LPS exposure in chronic obesity and SO remains uncertain. Therefore, intestinal lymphatic dysfunction may influence gut-derived inflammatory signaling, but this pathway requires direct validation in SO-specific models or patients.

Skeletal muscle repair is another potential but insufficiently validated pathway. Successful repair requires timely inflammation resolution, coordinated immune-cell transitions, muscle stem cell activation, differentiation, and fusion (73–75). In the context of obesity- or insulin-resistance-related metabolic stress, these repair processes may be impaired, leading to delayed regeneration and incomplete functional recovery (76). The lymphatic system may support this process by regulating tissue fluid homeostasis, inflammatory mediator clearance, metabolic waste drainage, and immune-cell trafficking, thereby contributing to the transition from a pro-inflammatory to a repair-supportive microenvironment (21, 77, 78).

The intramuscular lymphatic network is particularly relevant to local repair microenvironmental renewal. Although lymphatic vessels in skeletal muscle are relatively sparse, emerging evidence suggests that they are dynamic structures that may remodel during unloading, injury, and early repair (23). Hindlimb unloading has been reported to reduce intramuscular lymphatic capillary density and downregulate VEGF-C/VEGF-D expression, whereas stretch-contraction-induced injury can promote intramuscular lymphatic expansion and morphological remodeling during early repair (79, 80). These findings suggest that intramuscular lymphatics may contribute to post-injury microenvironmental renewal by facilitating interstitial fluid clearance, inflammatory mediator removal, metabolic waste drainage, and immune-cell trafficking. In SO, ageing-related reductions in lymphatic reserve may interact with obesity-associated inflammation, lipotoxicity, and insulin resistance to create a repair-unfavorable skeletal muscle milieu, but this possibility remains to be tested directly. Therefore, both the gut–lymphatic–muscle axis and intramuscular lymphatic remodeling should be regarded as plausible but unproven directions for future SO-specific investigation.

### 4.4 SO-specific mechanistic evidence gaps

Several SO-specific mechanistic evidence gaps remain. First, it is unknown whether patients or animal models with SO exhibit measurable lymphatic abnormalities beyond those attributable to ageing or obesity alone. This gap requires comparisons between SO and age- and obesity-matched non-sarcopenic controls using lymphatic imaging, tracer clearance assays, permeability assessment, collecting-vessel pumping measurements, macromolecular clearance analysis, and lymphatic marker profiling.

Second, the inflammatory clearance pathway requires direct validation in SO. Although impaired lymphatic drainage may delay inflammatory mediator clearance and inflammation resolution, it remains unclear whether lymphatic dysfunction in SO is associated with persistent retention of inflammatory mediators, immune-cell accumulation, delayed inflammation resolution, or sustained activation of catabolic inflammatory pathways in skeletal muscle.

Third, the lipid accumulation and insulin-resistance pathway also remains incompletely defined in SO. Existing evidence links lymphatic dysfunction more directly to adipose tissue expansion, lipid handling abnormalities, and lymphedema-associated fat deposition than to skeletal muscle lipid infiltration. It remains unclear whether impaired lymphatic clearance is associated with intramuscular lipid accumulation, intermuscular adipose tissue expansion, insulin-signaling defects, or reduced muscle strength and physical performance in SO.

Fourth, the gut–lymphatic–muscle axis and skeletal muscle repair microenvironment require further SO-specific validation. Current evidence for these pathways is mainly extrapolated from intestinal lymphatic biology, microbiota studies, muscle injury, unloading, and general lymphatic models rather than SO-specific settings. Whether mesenteric, visceral adipose, and intramuscular lymphatic abnormalities directly contribute to skeletal muscle metabolic stress or defective regeneration in SO remains uncertain.

Finally, no SO-specific intervention study has tested whether restoring lymphatic drainage, barrier integrity, or interstitial clearance can improve skeletal muscle outcomes. It also remains unclear whether lymphatic dysfunction is a contributor to SO progression, a consequence of skeletal muscle deterioration, or part of a bidirectional feed-forward loop involving reduced contraction-assisted lymphatic return.

## 5 Intervention strategies targeting lymphatic function

Based on this framework, lymphatic-targeted interventions should not be interpreted as established treatments for SO. Their current relevance lies in the hypothesis that improving lymphatic drainage, barrier integrity, or interstitial microenvironmental renewal may reduce the persistence of inflammatory and metabolic stressors acting on adipose tissue and skeletal muscle. Because direct SO-specific and human evidence remains limited, the following strategies are discussed as exploratory adjunctive approaches rather than clinically validated therapies.

### 5.1 Lifestyle interventions: fat reduction, muscle preservation, and promotion of lymphatic return

Lifestyle intervention should be considered a practical entry point in SO-related prevention and management, although it is not a lymphatic-specific therapy. Weight control, resistance training, aerobic exercise, and nutritional support may reduce adipose-driven inflammatory and metabolic stress while helping to preserve skeletal muscle function. Among these strategies, resistance training is particularly relevant because it improves muscle mass, strength, body composition, and physical performance (81–83), and repeated skeletal muscle contraction may also support lymphatic return through a muscle-pump effect (84, 85). Lifestyle intervention may therefore have potential lymphatic relevance in addition to its established metabolic and musculoskeletal benefits. In animal models of obesity, weight loss has been shown to reverse obesity-induced lymphatic dysfunction, while aerobic exercise can improve lymphatic function even independently of weight loss (86, 87). These findings support the rationale for considering lifestyle intervention as a lymphatic-supportive strategy in obesity-related conditions, although direct evidence in SO remains lacking. Nutritional support also remains important for maintaining muscle anabolism, supporting exercise adaptation, and limiting lean mass loss during weight reduction.

### 5.2 Supportive lymphatic interventions and molecular targeting strategies

Among supportive lymphatic approaches, manual lymphatic drainage provides a useful example of how improving lymph flow may have potential effects on metabolic and inflammatory outcomes, but its relevance to SO should be interpreted cautiously. Existing studies have reported improvements in glucose and lipid metabolic parameters, insulin resistance-related markers, and quality of life in individuals with abnormal body weight after manual lymphatic drainage (88–90). These findings are consistent with the idea that enhanced lymphatic return may help support the removal of inflammatory and metabolic by-products. The available studies, however, were not designed specifically for SO, and their sample sizes, intervention protocols, and outcome measures remain heterogeneous. Manual lymphatic drainage should therefore be regarded as an exploratory supportive strategy rather than a validated SO-specific intervention.

At the molecular level, the VEGF-C/D–VEGFR3 axis represents a central regulator of lymphangiogenesis and lymphatic repair, but its translational interpretation in obesity and SO should remain cautious. Existing evidence suggests that both activation and inhibition of this pathway may improve obesity-related metabolic abnormalities under different experimental conditions (91, 92), indicating that the outcome probably depends on tissue context, disease stage, inflammatory status, and the quality of newly formed lymphatic vessels. Current clinical experience with lymphatic repair is derived mainly from secondary lymphedema rather than SO. For example, VEGF-C gene therapy with Lymfactin^®^ has shown favorable safety in phase I/II studies and suggested possible improvements in lymphatic drainage-related outcomes in secondary lymphedema (93–95). These findings support the clinical feasibility of restoring lymphatic drainage, but evidence for lymphatic-targeted therapy in SO remains lacking.

Modulating the inflammatory-metabolic environment surrounding lymphatic vessels represents another potential direction beyond lymphangiogenesis and structural lymphatic repair. For example, in diet-induced obesity, COX-2/PGE_2_ signaling, together with VEGF-C–VEGFR3 signaling, promotes abnormal mesenteric lymphatic branching and leakage of high-fat-diet-modified lymph into visceral adipose tissue, thereby contributing to visceral adipose inflammation and insulin resistance (12). Inflammatory lipid mediators such as LTB4 may also represent candidate pathways, although current evidence is mainly derived from obesity-related insulin resistance and lymphedema models rather than SO-specific studies (96, 97).

Improving lymphatic function may require not only promoting lymphatic growth, but also reducing the inflammatory and metabolic stress that impairs lymphatic transport. GLP-1 receptor agonists may be relevant in this context because of their effects on body weight, metabolic inflammation, and adipose tissue dysfunction, although direct evidence for lymphatic improvement in SO remains lacking. Future studies should determine whether lymphatic restoration can be integrated with fat reduction, metabolic improvement, and muscle preservation in SO-specific settings.

## 6 Limitations and future experimental validation

A major limitation of the proposed framework is the lack of direct human and SO-specific experimental evidence linking lymphatic dysfunction to sarcopenic obesity. Most available evidence is derived from obesity models, ageing-related lymphatic studies, lymphedema, diabetes, intestinal lymphatic biology, or experimental muscle injury, rather than from patients or animal models specifically designed to study SO. Therefore, the proposed clearance-centered model should be interpreted as a hypothesis-generating framework rather than an established causal pathway. Although the main SO-specific mechanistic evidence gaps are summarized in Section 4.4, future studies will require longitudinal, mechanistic, and interventional designs to determine whether lymphatic dysfunction precedes, accompanies, or follows skeletal muscle deterioration, and whether restoring lymphatic drainage or interstitial clearance can meaningfully improve muscle outcomes in SO-specific settings.

## 7 Conclusion and future perspectives

Current evidence is consistent with a biologically plausible framework in which lymphatic dysfunction may help link ageing- and obesity-associated tissue alterations to skeletal muscle deterioration by altering the interstitial environment in which adipose tissue and skeletal muscle interact. Rather than acting as a single initiating cause of SO, impaired lymphatic clearance may prolong local exposure to retained inflammatory and metabolic stressors, thereby reinforcing a hostile tissue milieu.

Within this framework, ageing and obesity may contribute to lymphatic dysfunction through partly distinct but convergent mechanisms. Ageing may lower baseline lymphatic reserve, whereas obesity increases inflammatory, lipid, and metabolic lymphatic burden. Their convergence may amplify SO-related tissue dysfunction by impairing interstitial clearance and microenvironmental renewal. As skeletal muscle mass and contractile function decline, however, contraction-assisted lymphatic return may also be weakened, which could further promote lymph stasis, impaired clearance, and inflammatory–metabolic signal retention. This reciprocal relationship suggests that lymphatic dysfunction and skeletal muscle deterioration may reinforce each other in a bidirectional feed-forward loop, amplifying rather than solely initiating SO progression.

This perspective does not replace the established adipose–muscle crosstalk model, but adds a clearance-centered dimension to it. In this view, the persistence of pathological signals within the interstitial compartment is as important as their production. The evidence base remains indirect because most available findings come from obesity, ageing, lymphedema, diabetes, intestinal lymphatic studies, or experimental muscle injury models rather than SO-specific settings. Future work should determine whether lymphatic dysfunction can be measured, modified, and functionally linked to muscle loss in SO-specific models and clinical populations, and whether interventions that restore lymphatic clearance can be integrated with strategies targeting adipose dysfunction, metabolic impairment, and muscle preservation.

## References

[1] Gao Q Mei F Shang Y Hu K Chen F Zhao L . Global prevalence of sarcopenic obesity in older adults: a systematic review and meta-analysis. Clin Nutr. (2021) 40:4633–41. doi: 10.1016/j.clnu.2021.06.009 34229269

[2] Wannamethee SG Atkins JL . Sarcopenic obesity and cardiometabolic health and mortality in older adults: a growing health concern in an ageing population. Curr Diabetes Rep. (2023) 23:307–14. doi: 10.1007/s11892-023-01522-2 37566368 PMC10640508

[3] Barazzoni R Bischoff SC Boirie Y Busetto L Cederholm T Dicker D . Sarcopenic obesity: time to meet the challenge. Clin Nutr. (2018) 37:1787–93. doi: 10.1016/j.clnu.2018.04.018 29857921

[4] Park MJ Choi KM . Interplay of skeletal muscle and adipose tissue: sarcopenic obesity. Metabolism. (2023) 144:155577. doi: 10.1016/j.metabol.2023.155577 37127228

[5] Prado CM Batsis JA Donini LM Gonzalez MC Siervo M . Sarcopenic obesity in older adults: a clinical overview. Nat Rev Endocrinol. (2024) 20:261. doi: 10.1038/s41574-023-00943-z 38321142 PMC12854800

[6] Hong S-H Choi KM . Sarcopenic obesity, insulin resistance, and their implications in cardiovascular and metabolic consequences. Int J Mol Sci. (2020) 21:494. doi: 10.3390/ijms21020494 31941015 PMC7013734

[7] Wiig H Swartz MA . Interstitial fluid and lymph formation and transport: physiological regulation and roles in inflammation and cancer. Physiol Rev. (2012) 92:1005–60. doi: 10.1152/physrev.00037.2011 22811424

[8] Schwager S Detmar M . Inflammation and lymphatic function. Front Immunol. (2019) 10:308. doi: 10.3389/fimmu.2019.00308 30863410 PMC6399417

[9] Jiang X Tian W Nicolls MR Rockson SG . The lymphatic system in obesity, insulin resistance, and cardiovascular diseases. Front Physiol. (2019) 10:1402. doi: 10.3389/fphys.2019.01402 31798464 PMC6868002

[10] De Nardo W Chan AY Porter CJH Cao E Trevaskis NL . Dysfunctional adipose tissue-lymphatic crosstalk in obesity. Nat Rev Endocrinol. (2026) 22:416–32. doi: 10.1038/s41574-026-01243-y 41840147

[11] Weitman ES Aschen SZ Farias-Eisner G Albano N Cuzzone DA Ghanta S . Obesity impairs lymphatic fluid transport and dendritic cell migration to lymph nodes. PloS One. (2013) 8:e70703. doi: 10.1371/journal.pone.0070703 23950984 PMC3741281

[12] Cao E Watt MJ Nowell CJ Quach T Simpson JS De Melo Ferreira V . Mesenteric lymphatic dysfunction promotes insulin resistance and represents a potential treatment target in obesity. Nat Metab. (2021) 3:1175–88. doi: 10.1038/s42255-021-00457-w 34545251

[13] Zolla V Nizamutdinova IT Scharf B Clement CC Maejima D Akl T . Aging-related anatomical and biochemical changes in lymphatic collectors impair lymph transport, fluid homeostasis, and pathogen clearance. Aging Cell. (2015) 14:582–94. doi: 10.1111/acel.12330 25982749 PMC4531072

[14] Da Mesquita S Louveau A Vaccari A Smirnov I Cornelison RC Kingsmore KM . Functional aspects of meningeal lymphatics in ageing and Alzheimer’s disease. Nature. (2018) 560:185–91. doi: 10.1038/s41586-018-0368-8 30046111 PMC6085146

[15] Yang Y Wang X Wang P . Signaling mechanisms underlying lymphatic vessel dysfunction in skin aging and possible anti-aging strategies. Biogerontology. (2023) 24:727–40. doi: 10.1007/s10522-023-10016-3 36680698

[16] Donini LM Busetto L Bischoff SC Cederholm T Ballesteros-Pomar MD Batsis JA . Definition and diagnostic criteria for sarcopenic obesity: ESPEN and EASO consensus statement. Obes Facts. (2022) 15:321–35. doi: 10.1159/000521241 35196654 PMC9210010

[17] Cruz-Jentoft AJ Bahat G Bauer J Boirie Y Bruyère O Cederholm T . Sarcopenia: revised European consensus on definition and diagnosis. Age Ageing. (2019) 48:16–31. doi: 10.1093/ageing/afy169 30312372 PMC6322506

[18] Kawai T Autieri MV Scalia R . Adipose tissue inflammation and metabolic dysfunction in obesity. Am J Physiol Cell Physiol. (2021) 320:C375–91. doi: 10.1152/ajpcell.00379.2020 33356944 PMC8294624

[19] Lu W Feng W Lai J Yuan D Xiao W Li Y . Role of adipokines in sarcopenia. Chin Med J (Engl). (2023) 136:1794–804. doi: 10.1097/CM9.0000000000002255 37442757 PMC10406092

[20] Johnson LA Jackson DG . Control of dendritic cell trafficking in lymphatics by chemokines. Angiogenesis. (2014) 17:335–45. doi: 10.1007/s10456-013-9407-0 24232855

[21] Johnson LA . In sickness and in health: the immunological roles of the lymphatic system. Int J Mol Sci. (2021) 22:4458. doi: 10.3390/ijms22094458 33923289 PMC8123157

[22] Havas E Parviainen T Vuorela J Toivanen J Nikula T Vihko V . Lymph flow dynamics in exercising human skeletal muscle as detected by scintography. J Physiol. (1997) 504:233–9. doi: 10.1111/j.1469-7793.1997.233bf.x 9350633 PMC1159951

[23] Ji R-C . Recent advances and new insights into muscular lymphangiogenesis in health and disease. Life Sci. (2018) 211:261–9. doi: 10.1016/j.lfs.2018.09.043 30261160

[24] Kataru RP Park HJ Baik JE Li C Shin J Mehrara BJ . Regulation of lymphatic function in obesity. Front Physiol. (2020) 11:459. doi: 10.3389/fphys.2020.00459 32499718 PMC7242657

[25] Sudduth CL Greene AK . Lymphedema and obesity. Cold Spring Harb Perspect Med. (2022) 12:a041176. doi: 10.1101/cshperspect.a041176 35074795 PMC9159261

[26] Torrisi JS Hespe GE Cuzzone DA Savetsky IL Nitti MD Gardenier JC . Inhibition of inflammation and iNOS improves lymphatic function in obesity. Sci Rep. (2016) 6:19817. doi: 10.1038/srep19817 26796537 PMC4726274

[27] Blum KS Karaman S Proulx ST Ochsenbein AM Luciani P Leroux J-C . Chronic high-fat diet impairs collecting lymphatic vessel function in mice. PloS One. (2014) 9:e94713. doi: 10.1371/journal.pone.0094713 24714646 PMC3979858

[28] García Nores GD Cuzzone DA Albano NJ Hespe GE Kataru RP Torrisi JS . Obesity but not high-fat diet impairs lymphatic function. Int J Obes. (2016) 40:1582–90. doi: 10.1038/ijo.2016.96 27200507 PMC5050064

[29] Cifarelli V Eichmann A . The intestinal lymphatic system: functions and metabolic implications. Cell Mol Gastroenterol Hepatol. (2018) 7:503–13. doi: 10.1016/j.jcmgh.2018.12.002 30557701 PMC6396433

[30] Mikrani R Styles IK Hoang TA Abdallah M Senyschyn D Porter CJH . Obesity-associated mesenteric lymph leakage impairs the trafficking of lipids, lipophilic drugs and antigens from the intestine to mesenteric lymph nodes. Eur J Pharm Biopharm Off J Arbeitsgemeinschaft Pharm Verfahrenstechnik Evol. (2022) 180:319–31. doi: 10.1016/j.ejpb.2022.10.019 36283633

[31] Cromer WE Zawieja SD Tharakan B Childs EW Newell MK Zawieja DC . The effects of inflammatory cytokines on lymphatic endothelial barrier function. Angiogenesis. (2014) 17:395–406. doi: 10.1007/s10456-013-9393-2 24141404 PMC4314095

[32] Chen Y Rehal S Roizes S Zhu H-L Cole WC von der Weid P-Y . The pro-inflammatory cytokine TNF-α inhibits lymphatic pumping via activation of the NF-κB-iNOS signaling pathway. Microcirculation. (2017) 24:e12364. doi: 10.1111/micc.12364 28231612 PMC5404961

[33] Mathias R von der Weid P-Y . Involvement of the NO-cGMP-K(ATP) channel pathway in the mesenteric lymphatic pump dysfunction observed in the Guinea pig model of TNBS-induced ileitis. Am J Physiol Gastrointest Liver Physiol. (2013) 304:G623–34. doi: 10.1152/ajpgi.00392.2012 23275612

[34] Rehal S Kataru RP Hespe GE Baik JE Park HJ Ly C . Regulation of lymphatic function and injury by nitrosative stress in obese mice. Mol Metab. (2020) 42:101081. doi: 10.1016/j.molmet.2020.101081 32941994 PMC7536739

[35] Kraft JD Blomgran R Lundgaard I Quiding-Järbrink M Bromberg JS Börgeson E . Specialized pro-resolving mediators and the lymphatic system. Int J Mol Sci. (2021) 22:2750. doi: 10.3390/ijms22052750 33803130 PMC7963193

[36] Redondo PA Gubert F Zaverucha-do-Valle C Dutra TPP Ayres-Silva JP Fernandes N . Lymphatic vessels in human adipose tissue. Cell Tissue Res. (2020) 379:511–20. doi: 10.1007/s00441-019-03108-5 31776824

[37] Xia Q Dong H Guo Y Fang K Hu M Xu L . The role of lacteal integrity and junction transformation in obesity: a promising therapeutic target? Front Endocrinol. (2022) 13:1007856. doi: 10.3389/fendo.2022.1007856 36506056 PMC9729342

[38] Lim HY Thiam CH Yeo KP Bisoendial R Hii CS McGrath KCY . Lymphatic vessels are essential for the removal of cholesterol from peripheral tissues by SR-BI-mediated transport of HDL. Cell Metab. (2013) 17:671–84. doi: 10.1016/j.cmet.2013.04.002 23663736

[39] Huang L-H Elvington A Randolph GJ . The role of the lymphatic system in cholesterol transport. Front Pharmacol. (2015) 6:182. doi: 10.3389/fphar.2015.00182 26388772 PMC4557107

[40] Zhang F Zarkada G Han J Li J Dubrac A Ola R . Lacteal junction zippering protects against diet-induced obesity. Science. (2018) 361:599–603. doi: 10.1126/science.aap9331 30093598 PMC6317738

[41] Tokarz VL Pereira RVS Jaldin-Fincati JR Mylvaganam S Klip A . Junctional integrity and directional mobility of lymphatic endothelial cell monolayers are disrupted by saturated fatty acids. Mol Biol Cell. (2023) 34:ar28. doi: 10.1091/mbc.E22-08-0367 36735487 PMC10092641

[42] Cifarelli V Appak-Baskoy S Peche VS Kluzak A Shew T Narendran R . Visceral obesity and insulin resistance associate with CD36 deletion in lymphatic endothelial cells. Nat Commun. (2021) 12:3350. doi: 10.1038/s41467-021-23808-3 34099721 PMC8184948

[43] Kataru RP Park HJ Shin J Baik JE Sarker A Brown S . Structural and functional changes in aged skin lymphatic vessels. Front Aging. (2022) 3:864860. doi: 10.3389/fragi.2022.864860 35821848 PMC9261401

[44] Tuckey B Srbely J Rigney G Vythilingam M Shah J . Impaired lymphatic drainage and interstitial inflammatory stasis in chronic musculoskeletal and idiopathic pain syndromes: exploring a novel mechanism. Front Pain Res. (2021) 2. doi: 10.3389/fpain.2021.691740 35295453 PMC8915610

[45] Sato A Kamekura R Kawata K Kawada M Jitsukawa S Yamashita K . Novel mechanisms of compromised lymphatic endothelial cell homeostasis in obesity: the role of leptin in lymphatic endothelial cell tube formation and proliferation. PloS One. (2016) 11:e0158408. doi: 10.1371/journal.pone.0158408 27366905 PMC4930203

[46] Castillo ÍMP Argilés JM Rueda R Ramírez M Pedrosa JML . Skeletal muscle atrophy and dysfunction in obesity and type-2 diabetes mellitus: myocellular mechanisms involved. Rev Endocr Metab Disord. (2025) 26:815–36. doi: 10.1007/s11154-025-09954-9 40064750 PMC12534344

[47] Sartori R Romanello V Sandri M . Mechanisms of muscle atrophy and hypertrophy: implications in health and disease. Nat Commun. (2021) 12:330. doi: 10.1038/s41467-020-20123-1 33436614 PMC7803748

[48] Souza A Alves ALR Martinez CG Sousa J Kurtenbach E . Biomarkers of skeletal muscle atrophy based on atrogenes evaluation: a systematic review and meta-analysis study. Int J Mol Sci. (2025) 26:3516. doi: 10.3390/ijms26083516 40331994 PMC12026492

[49] Karkkainen MJ Saaristo A Jussila L Karila KA Lawrence EC Pajusola K . A model for gene therapy of human hereditary lymphedema. Proc Natl Acad Sci USA. (2001) 98:12677–82. doi: 10.1073/pnas.221449198 11592985 PMC60113

[50] Harvey NL Srinivasan RS Dillard ME Johnson NC Witte MH Boyd K . Lymphatic vascular defects promoted by Prox1 haploinsufficiency cause adult-onset obesity. Nat Genet. (2005) 37:1072–81. doi: 10.1038/ng1642 16170315

[51] Lee S-O Kim I-K . Molecular pathophysiology of secondary lymphedema. Front Cell Dev Biol. (2024) 12:1363811. doi: 10.3389/fcell.2024.1363811 39045461 PMC11264244

[52] Hsiao H-Y Liu J-W Pappalardo M Cheng M-H . The impacts of lymph on the adipogenesis of adipose-derived stem cells. Plast Reconstr Surg. (2023) 151:1005–15. doi: 10.1097/PRS.0000000000010082 36534068

[53] Escobedo N Oliver G . The lymphatic vasculature: its role in adipose metabolism and obesity. Cell Metab. (2017) 26:598–609. doi: 10.1016/j.cmet.2017.07.020 28844882 PMC5629116

[54] Savetsky IL Torrisi JS Cuzzone DA Ghanta S Albano NJ Gardenier JC . Obesity increases inflammation and impairs lymphatic function in a mouse model of lymphedema. Am J Physiol Heart Circ Physiol. (2014) 307:H165–172. doi: 10.1152/ajpheart.00244.2014 24858842 PMC4101643

[55] Ingram KH Lara-Castro C Gower BA Makowsky R Allison DB Newcomer BR . Intramyocellular lipid and insulin resistance: differential relationships in European and African Americans. Obesity. (2011) 19:1469–75. doi: 10.1038/oby.2011.45 21436797 PMC3171736

[56] Choi SJ Files DC Zhang T Wang Z-M Messi ML Gregory H . Intramyocellular lipid and impaired myofiber contraction in normal weight and obese older adults. J Gerontol A Biol Sci Med Sci. (2016) 71:557–64. doi: 10.1093/gerona/glv169 26405061 PMC5014190

[57] Sachs S Zarini S Kahn DE Harrison KA Perreault L Phang T . Intermuscular adipose tissue directly modulates skeletal muscle insulin sensitivity in humans. Am J Physiol Endocrinol Metab. (2019) 316:E866–79. doi: 10.1152/ajpendo.00243.2018 30620635 PMC6580171

[58] Goodpaster BH Bergman BC Brennan AM Sparks LM . Intermuscular adipose tissue in metabolic disease. Nat Rev Endocrinol. (2023) 19:285–98. doi: 10.1038/s41574-022-00784-2 36564490

[59] Kitessa SM Abeywardena MY . Lipid-induced insulin resistance in skeletal muscle: the chase for the culprit goes from total intramuscular fat to lipid intermediates, and finally to species of lipid intermediates. Nutrients. (2016) 8:466. doi: 10.3390/nu8080466 27483311 PMC4997379

[60] Szendroedi J Yoshimura T Phielix E Koliaki C Marcucci M Zhang D . Role of diacylglycerol activation of PKCθ in lipid-induced muscle insulin resistance in humans. Proc Natl Acad Sci USA. (2014) 111:9597–602. doi: 10.1073/pnas.1409229111 24979806 PMC4084449

[61] Bandet CL Tan-Chen S Bourron O Le Stunff H Hajduch E . Sphingolipid metabolism: new insight into ceramide-induced lipotoxicity in muscle cells. Int J Mol Sci. (2019) 20:479. doi: 10.3390/ijms20030479 30678043 PMC6387241

[62] van Gerwen J Shun-Shion AS Fazakerley DJ . Insulin signalling and GLUT4 trafficking in insulin resistance. Biochem Soc Trans. (2023) 51:1057–69. doi: 10.1042/BST20221066 37248992 PMC10317183

[63] Abdul-Ghani MA DeFronzo RA . Pathogenesis of insulin resistance in skeletal muscle. J BioMed Biotechnol. (2010) 2010:476279. doi: 10.1155/2010/476279 20445742 PMC2860140

[64] Schiaffino S Mammucari C . Regulation of skeletal muscle growth by the IGF1-Akt/PKB pathway: insights from genetic models. Skelet Muscle. (2011) 1:4. doi: 10.1186/2044-5040-1-4 21798082 PMC3143906

[65] Sandri M Sandri C Gilbert A Skurk C Calabria E Picard A . Foxo transcription factors induce the atrophy-related ubiquitin ligase atrogin-1 and cause skeletal muscle atrophy. Cell. (2004) 117:399–412. doi: 10.1016/s0092-8674(04)00400-3 15109499 PMC3619734

[66] Li T Yin D Shi R . Gut-muscle axis mechanism of exercise prevention of sarcopenia. Front Nutr. (2024) 11:1418778. doi: 10.3389/fnut.2024.1418778 39221163 PMC11362084

[67] Wang L He X Zhang Z Chen N . Distinct gut microbiota signatures in older people with sarcopenic obesity and sarcopenia without obesity. Clin Nutr. (2025) 49:77–89. doi: 10.1016/j.clnu.2025.04.004 40252601

[68] Suh SH Choe K Hong SP Jeong S-H Mäkinen T Kim KS . Gut microbiota regulates lacteal integrity by inducing VEGF-C in intestinal villus macrophages. EMBO Rep. (2019) 20:e46927. doi: 10.15252/embr.201846927 30783017 PMC6446200

[69] Akiba Y Maruta K Takajo T Narimatsu K Said H Kato I . Lipopolysaccharides transport during fat absorption in rodent small intestine. Am J Physiol Gastrointest Liver Physiol. (2020) 318:G1070–87. doi: 10.1152/ajpgi.00079.2020 32390462 PMC7311662

[70] Ghoshal S Witta J Zhong J de Villiers W Eckhardt E . Chylomicrons promote intestinal absorption of lipopolysaccharides. J Lipid Res. (2009) 50:90–7. doi: 10.1194/jlr.M800156-JLR200 18815435

[71] Doyle A Zhang G Abdel Fattah EA Eissa NT Li Y-P . Toll-like receptor 4 mediates lipopolysaccharide-induced muscle catabolism via coordinate activation of ubiquitin-proteasome and autophagy-lysosome pathways. FASEB J Off Publ Fed Am Soc Exp Biol. (2011) 25:99–110. doi: 10.1096/fj.10-164152 20826541 PMC3005430

[72] Liang H Hussey SE Sanchez-Avila A Tantiwong P Musi N . Effect of lipopolysaccharide on inflammation and insulin action in human muscle. PloS One. (2013) 8:e63983. doi: 10.1371/journal.pone.0063983 23704966 PMC3660322

[73] Wang X Zhou L . The many roles of macrophages in skeletal muscle injury and repair. Front Cell Dev Biol. (2022) 10:952249. doi: 10.3389/fcell.2022.952249 35898401 PMC9309511

[74] Rigamonti E Zordan P Sciorati C Rovere-Querini P Brunelli S . Macrophage plasticity in skeletal muscle repair. BioMed Res Int. (2014) 2014:560629. doi: 10.1155/2014/560629 24860823 PMC4016840

[75] Yang W Hu P . Skeletal muscle regeneration is modulated by inflammation. J Orthop Transl. (2018) 13:25–32. doi: 10.1016/j.jot.2018.01.002 29662788 PMC5892385

[76] Espino-Gonzalez E Dalbram E Mounier R Gondin J Farup J Jessen N . Impaired skeletal muscle regeneration in diabetes: from cellular and molecular mechanisms to novel treatments. Cell Metab. (2024) 36:1204–36. doi: 10.1016/j.cmet.2024.02.014 38490209

[77] Oliver G Kipnis J Randolph GJ Harvey NL . The lymphatic vasculature in the 21st century: novel functional roles in homeostasis and disease. Cell. (2020) 182:270–96. doi: 10.1016/j.cell.2020.06.039 32707093 PMC7392116

[78] Liao S von der Weid P-Y . Lymphatic system: an active pathway for immune protection. Semin Cell Dev Biol. (2015) 38:83–9. doi: 10.1016/j.semcdb.2014.11.012 25534659 PMC4397130

[79] Tamura Y Kawashima T Ji R-C Agata N Itoh Y Kawakami K . Histological and biochemical changes in lymphatic vessels after skeletal muscle injury induced by lengthening contraction in male mice. Physiol Rep. (2024) 12:e15950. doi: 10.14814/phy2.15950 38355142 PMC10866689

[80] Kawashima T Ji R-C Itoh Y Agata N Sasai N Murakami T . Morphological and biochemical changes of lymphatic vessels in the soleus muscle of mice after hindlimb unloading. Muscle Nerve. (2021) 64:620–8. doi: 10.1002/mus.27402 34409627

[81] Bilski J Pierzchalski P Szczepanik M Bonior J Zoladz JA . Multifactorial mechanism of sarcopenia and sarcopenic obesity. Role of physical exercise, microbiota and myokines. Cells. (2022) 11:160. doi: 10.3390/cells11010160 35011721 PMC8750433

[82] Eglseer D Traxler M Schoufour JD Weijs PJM Voortman T Boirie Y . Nutritional and exercise interventions in individuals with sarcopenic obesity around retirement age: a systematic review and meta-analysis. Nutr Rev. (2023) 81:1077–90. doi: 10.1093/nutrit/nuad007 36882046 PMC10413430

[83] Polo-Ferrero L Navarro-López V Fuentes M Lacal J Cancelas-Felgueras MD Santos-Blázquez N . Effect of resistance training on older adults with sarcopenic obesity: a comprehensive systematic review and meta-analysis of blood biomarkers, functionality, and body composition. Nurs Rep. (2025) 15:89. doi: 10.3390/nursrep15030089 40137662 PMC11944422

[84] Annunziata G Paoli A Manzi V Camajani E Laterza F Verde L . The role of physical exercise as a therapeutic tool to improve lipedema: a consensus statement from the Italian Society of Motor and Sports Sciences (Società Italiana di Scienze Motorie e Sportive, SISMeS) and the Italian Society of Phlebology (Società Italiana di Flebologia, SIF). Curr Obes Rep. (2024) 13:667–79. doi: 10.1007/s13679-024-00579-8 38958868 PMC11522091

[85] Shinaoka A Kimata Y . Lymphatic flow dynamics under exercise load assessed with thoracic duct ultrasonography. Sci Rep. (2025) 15:14323. doi: 10.1038/s41598-025-99416-8 40275043 PMC12022353

[86] Nitti MD Hespe GE Kataru RP García Nores GD Savetsky IL Torrisi JS . Obesity-induced lymphatic dysfunction is reversible with weight loss. J Physiol. (2016) 594:7073–87. doi: 10.1113/JP273061 27619475 PMC5134379

[87] Hespe GE Kataru RP Savetsky IL García Nores GD Torrisi JS Nitti MD . Exercise training improves obesity-related lymphatic dysfunction. J Physiol. (2016) 594:4267–82. doi: 10.1113/JP271757 26931178 PMC4967732

[88] Müller M Klingberg K Wertli MM Carreira H . Manual lymphatic drainage and quality of life in patients with lymphoedema and mixed oedema: a systematic review of randomised controlled trials. Qual Life Res Int J Qual Life Asp Treat Care Rehabil. (2018) 27:1403–14. doi: 10.1007/s11136-018-1796-5 29404923 PMC5951867

[89] Antoniak-Pietrynczak K Zorena K Jaskulak M Hansdorfer-Korzon R Koziński M . Effect of manual lymphatic drainage on the concentrations of selected adipokines, cytokines, C-reactive protein and parameters of carbohydrate and lipid metabolism in patients with abnormal body mass index: focus on markers of obesity and insulin resistance. Int J Mol Sci. (2023) 24:10338. doi: 10.3390/ijms241210338 37373485 PMC10299205

[90] Antoniak K Zorena K Hansdorfer-Korzon R Wojtowicz D Koziński M . Favourable changes in C-peptide, C-reactive protein and lipid profile, and improved quality of life in patients with abnormal body mass index after the use of manual lymphatic drainage: a case series with three-month follow-up. Med (Mex). (2022) 58:273. doi: 10.3390/medicina58020273 35208596 PMC8878077

[91] Chakraborty A Barajas S Lammoglia GM Reyna AJ Morley TS Johnson JA . Vascular endothelial growth factor-D (VEGF-D) overexpression and lymphatic expansion in murine adipose tissue improves metabolism in obesity. Am J Pathol. (2019) 189:924–39. doi: 10.1016/j.ajpath.2018.12.008 30878136 PMC6458523

[92] Karaman S Hollmén M Robciuc MR Alitalo A Nurmi H Morf B . Blockade of VEGF-C and VEGF-D modulates adipose tissue inflammation and improves metabolic parameters under high-fat diet. Mol Metab. (2015) 4:93–105. doi: 10.1016/j.molmet.2014.11.006 25685697 PMC4314545

[93] Hartiala P Suominen S Suominen E Kaartinen I Kiiski J Viitanen T . Phase 1 LymfactinⓇ study: short-term safety of combined adenoviral VEGF-C and lymph node transfer treatment for upper extremity lymphedema. J Plast Reconstr Aesthetic Surg JPRAS. (2020) 73:1612–21. doi: 10.1016/j.bjps.2020.05.009 32513642

[94] Leppäpuska I-M Hartiala P Suominen S Suominen E Kaartinen I Mäki M . Phase 1 Lymfactin® study: 24-month efficacy and safety results of combined adenoviral VEGF-C and lymph node transfer treatment for upper extremity lymphedema. J Plast Reconstr Aesthetic Surg JPRAS. (2022) 75:3938–45. doi: 10.1016/j.bjps.2022.08.011 36151039

[95] Rannikko EH Pajula S Suominen SH Kiiski J Mani MR Halle M . Phase II study shows the effect of adenoviral vascular endothelial growth factor C and lymph node transfer in lymphedema. Plast Reconstr Surg. (2025) 155:256e–67e. doi: 10.1097/PRS.0000000000011675 39137430

[96] Callegari IOM Oliveira AG . The role of LTB4 in obesity-induced insulin resistance development: an overview. Front Endocrinol. (2022) 13:848006. doi: 10.3389/fendo.2022.848006 35392132 PMC8981522

[97] Tian W Rockson SG Jiang X Kim J Begaye A Shuffle EM . Leukotriene B4 antagonism ameliorates experimental lymphedema. Sci Transl Med. (2017) 9:eaal3920. doi: 10.1126/scitranslmed.aal3920 28490670

